# Spinal Cord Stimulation Real-World Outcomes: A 24-Month Longitudinal Cohort Study

**DOI:** 10.3390/diagnostics15243149

**Published:** 2025-12-11

**Authors:** Giuliano Lo Bianco, Alexandra Therond, Francesco Paolo D’Angelo, Leonardo Kapural, Sudhir Diwan, Sean Li, Paul J. Christo, Jamal Hasoon, Timothy R. Deer, Christopher L. Robinson

**Affiliations:** 1 Anesthesiology and Pain Department, Foundation G. Giglio Cefalù, Via Pietrapollastra, 90015 Cefalù, Italy; 2 Department of Psychology, Université du Québec à Montréal, Montréal, QC H3C 3P8, Canada; therond.alexandra@courrier.uqam.ca; 3 Department of Anaesthesia, Intensive Care and Emergency, University Hospital Policlinico Paolo Giaccone, 90127 Palermo, Italy; francescods.dangelo@gmail.com; 4 Carolinas Pain Institute, Winston-Salem, NC 27103, USA; lkapuralmd@gmail.com; 5 Albert Einstein College of Medicine, Bronx, NY 10461, USA; sudhir.diwan63@gmail.com; 6 National Spine and Pain Centers, Shrewsbury, NJ 07702, USA; sli@treatingpain.com; 7 Division of Pain Medicine, Department of Anesthesiology and Critical Care Medicine, Johns Hopkins University School of Medicine, Baltimore, MD 21287, USA; pchristo@jhmi.edu; 8 Department of Anesthesiology, Critical Care, and Pain Medicine, The University of Texas Health Science Center at Houston, Houston, TX 77030, USA; jamal.j.hasoon@uth.tmc.edu; 9 The Spine and Nerve Centers of the Virginias, Charleston, WV 25301, USA; doctdeer@aol.com

**Keywords:** spinal cord stimulation, chronic pain, persistent spinal pain syndrome, neuropathic pain, real-world evidence, longitudinal cohort study, patient selection

## Abstract

**Background/Objectives:** Spinal cord stimulation (SCS) is an established therapy for chronic pain, but uncertainties remain regarding long-term real-world outcomes and the role of standardized selection pathways. This study aimed to evaluate real-world, longitudinal outcomes of SCS over 24 months within a structured clinical pathway, focusing on pain intensity, neuropathic symptoms, and health-related quality of life. **Methods:** A single-center, retrospective observational cohort study was conducted at the Fondazione Istituto G. Giglio (Cefalù, Italy). Data were drawn from the continuing, prospective institutional “SCS Pathway” and included consecutive patients implanted between May 2021 and September 2024. Eligible patients were ≥18 years of age with chronic pain refractory to conventional medical management. Outcomes included pain intensity (VAS, visual analog scale), neuropathic features (DN4, douleur neuropathique 4), and health-related quality of life (EQ-5D, EuroQol 5 Dimensions), assessed at baseline and 3, 6, 12, 18, and 24 months post-implantation. Multilevel models with full information maximum likelihood (FIML) were applied to repeated measures. **Results:** Seventy-six patients were included (mean age 67.3 ± 10.3 years; 39.5% female). The most frequent diagnoses were post-surgical pain syndrome (42.1%, 32/76) and chronic back and leg pain (40.8%, 31/76). 42.1% (32/76) had previous spine surgery, and 78.9% (60/76) reported neuropathic pain. Across 452 observations, mean VAS scores decreased from 7.9 ± 0.7 at baseline to 3.1 ± 1.1 at 3 months (61% reduction, *p* < 0.001), with sustained benefit at 24 months (4.5 ± 1.5; 43% reduction, *p* < 0.001). DN4 scores improved from 7.4 ± 0.8 to 3.2 ± 1.0 at 3 months (56% reduction, *p* < 0.001), with persistent decreases at 24 months (4.2 ± 1.2; 43% reduction, *p* < 0.001). EQ-5D improved from 22.8 ± 6.6 at baseline to 70.2 ± 10.6 at 3 months (increase of 208%, *p* < 0.001), with clinically meaningful gains sustained at 24 months (55.4 ± 13.7, increase of 143%, *p* < 0.001). **Conclusions:** In this real-world cohort, SCS therapy results in sustained, clinically significant improvements in pain, neuropathic symptoms, and quality of life. Findings highlight the value of structured selection and follow-up pathways. These data provide a benchmark for multicenter studies linking standardized referral frameworks to long-term, patient-centered outcomes.

## 1. Introduction

Spinal cord stimulation (SCS) is an established therapy for chronic neuropathic and mixed-mechanism pain conditions, including persistent spinal pain syndrome, chronic back and leg pain, and complex regional pain syndrome [1,2,3,4]. Since its first clinical application in 1967 by Shealy et al., who reported dorsal column stimulation for pain relief [5], SCS has undergone remarkable technological evolution. Early devices were limited by bulky hardware and narrow indications, whereas current systems offer paresthesia-free waveforms, including burst and high-frequency stimulation (10 kHz) [6], and more recently closed-loop technologies that adapt output in real-time to maintain efficacy [7].

Chronic pain itself represents a major global health burden, with prevalence estimates ranging from 19% to 30% in the adult population [8]. Neuropathic pain syndromes, in particular, are associated with higher disability, reduced quality of life, and increased healthcare costs compared to non-neuropathic conditions [9]. Despite this, registry data suggest SCS remains underutilized, with large geographical variations across Europe and North America [10]. Bridging this gap between evidence-based recommendations and real-world practice requires robust observational studies such as the present one.

Although randomized trials [6,11,12], international guidelines [12,13,14,15], and real-world evidence [16,17,18,19] support SCS therapy, patient selection remains pivotal. Contemporary recommendations emphasize integrating biomedical and psychosocial factors (e.g., pain phenotype, prior treatments, and psychological readiness) to optimize benefit and minimize risk [13,14,15,16,17,20,21,22]. To reduce variability across centers, structured methods and tools, such as the RAND/UCLA Appropriateness Method and a European e-Health instrument, have been developed to standardize referral criteria and improve transparency in selection [16,17,19,23,24]. Despite these advances, uncertainties persist in practice: which patients achieve durable improvement in pain and quality of life and how do real-world outcomes evolve beyond the early post-implant period? [25,26,27].

Beyond efficacy demonstrated in randomized trials [6,11], the real-world adoption of SCS has been shaped by several barriers. These include variability in referral practices, limited patient awareness, and the lack of harmonized multidisciplinary pathways across regions [16,17,19]. Epidemiological studies suggest that despite robust evidence, SCS remains underutilized compared to the prevalence of refractory neuropathic pain syndromes, with significant geographical differences in implantation rates across Europe and the United States [16,17,18,19].

From a pathophysiological standpoint, SCS exerts its effect through multiple mechanisms: modulation of dorsal column pathways according to the classical gate control theory, alterations in wide dynamic range neuronal activity, and potential changes in descending inhibitory circuits [1,2,3]. More recently, preclinical models have suggested that neuromodulation may influence glial activation and neuroinflammatory cascades, further supporting its role in complex, centralized pain states [11,19].

In addition, health economic analyses consistently demonstrate that SCS is cost-effective for selected populations, reducing healthcare utilization and indirect costs related to chronic pain disability [11,18,28]. However, these benefits depend on proper candidate identification and long-term adherence to follow-up protocols [17,21,22,23,24]. Standardized selection frameworks and digital decision-support systems, including emerging artificial intelligence applications, may contribute to minimizing inappropriate referrals and ensuring timely access to therapy [16,22,29,30]. Such integration of clinical expertise with structured pathways provides an opportunity to translate trial-level efficacy into durable, real-world effectiveness [19,28]. The present study aimed to quantify real-world clinical outcomes of SCS over 24 months within a standardized selection and follow-up pathway, evaluating pain intensity, neuropathic symptoms, and quality of life.

This analysis was designed to inform selection practices and to provide a benchmark for future work linking standardized referral criteria to long-term clinical benefit in real-world practice.

## 2. Methods

This introductory paragraph provides an overview of the study framework, while the subsequent subsections describe the design, data collection procedures, and statistical analysis in detail. This single-center, retrospective observational cohort study assessed the longitudinal effectiveness of spinal cord stimulation (SCS) in a real-world clinical pathway by quantifying changes in pain intensity (VAS, visual analog scale), neuropathic pain (DN4, douleur neuropathique 4), and health-related quality of life (EQ-5D, EuroQol 5 Dimensions) over a 24-month follow-up period using multilevel models (MLMs).

### 2.1. Study Design

This was a single-center, retrospective observational cohort study conducted at the Fondazione Istituto G. Giglio (Cefalù, Palermo, Italy). Data were drawn from the prospectively managed institutional “SCS Pathway,” which standardizes evaluation and follow-up for candidates undergoing spinal cord stimulation (SCS). Patients were consecutively enrolled between 1 May 2021 and 30 September 2024. Inclusion criteria comprised chronic pain syndromes refractory to conventional medical management, including persistent spinal pain syndrome, chronic back and leg pain, complex regional pain syndrome, and neuropathic pain. Exclusion criteria were uncontrolled psychiatric comorbidity, active systemic infection, or contraindications to implantation (e.g., uncorrected coagulopathy, inability to undergo anesthesia or surgery, severe cognitive impairment precluding informed consent, or active substance use disorder). All participants were ≥18 years of age and evaluated by a multidisciplinary team (MDT) composed of two pain physicians, a psychologist, and a specialist nurse. The MDT met in case conferences and reached decisions about SCS candidacy by consensus. Disagreements were resolved through discussion, and patients were not offered a trial unless all MDT members considered the risk–benefit profile acceptable and modifiable psychosocial factors had been addressed. The MDT assessment integrated biomedical and psychosocial factors to determine candidacy for SCS trialing and implantation. Although the SCS Pathway is conceptually aligned with the RAND/UCLA Appropriateness Method and the European e-health referral tool, these instruments were not applied in a formal scoring manner in this cohort. Instead, their criteria informed local MDT-based decisions and contributed to a structured, transparent selection process.

The institutional “SCS Pathway” consists of several standardized phases. After initial referral, patients undergo a comprehensive assessment that includes pain history, neurological examination, and targeted imaging when indicated. This pathway is grounded in European consensus recommendations and local clinical policies, and is designed to ensure consistent referral, multimodal evaluation, and longitudinal follow-up. At each step, standardized clinical forms are completed and stored in the electronic health record, allowing traceability of multidisciplinary decisions and outcomes. Psychological evaluation is mandatory and focuses on screening for depression, anxiety, catastrophizing, and readiness for implantable therapies.

Standardized instruments included the Hospital Anxiety and Depression Scale (HADS) and the Pain Catastrophizing Scale (PCS), both validated for chronic pain populations [31,32,33]. These tools not only excluded uncontrolled psychiatric conditions but also identified psychosocial predictors of poor outcome, in line with international consensus [16]. The HADS and PCS questionnaires were administered as part of the routine psychological assessment to exclude uncontrolled psychiatric conditions and to identify psychosocial risk factors. However, individual questionnaire scores were not collected in analyzable form for research purposes and were therefore not included in the statistical analyses, because the psychological assessment was used solely for clinical screening purposes within the SCS Pathway and not for research analysis.

The study was conceived and conducted at the Fondazione Istituto G. Giglio. International co-authors based in Canada and the United States contributed to the study design, statistical analysis plan, interpretation of findings, and drafting and critical revision of the manuscript, particularly with regard to methodological and neuromodulation expertise. Although the cohort included different chronic pain etiologies, all were neuropathic or mixed-mechanism pain syndromes recognized as appropriate indications for SCS according to international guidelines (NICE, NeuPSIG, European consensus). These diagnoses represent the typical clinical spectrum referred for neuromodulation in real-world settings.

### 2.2. Trial Stimulation, Implantation, and Device Management

Trial stimulation was performed under fluoroscopic guidance with percutaneous cylindrical leads, typically lasting 7–14 days. During this period, programming was individualized to optimize paresthesia coverage, or in the case of 10 kHz high-frequency systems, to achieve pain relief without sensory perception. Success was defined as ≥50% reduction in pain intensity or meaningful functional improvement, evaluated through daily patient diaries and multidisciplinary team (MDT) review. Permanent implantation was performed exclusively in patients who met these predefined success criteria during the trial. Daily diaries were paper-based and captured average daily pain intensity (0–10 scale), sleep quality, and simple functional markers (e.g., walking tolerance, basic activities). Patients completed these diaries at home and brought them to the clinic visit at the end of the trial. In routine practice, adherence to diary completion was high and the MDT systematically reviewed diary trends alongside in-person clinical assessment to determine whether predefined success criteria were met. Only successful candidates proceeded to permanent implantation. This structured approach aligns with European consensus and recent randomized data supporting the value of screening trials [16,28].

Regarding device characteristics, both conventional low-frequency and high-frequency systems from multiple commercial manufacturers were available at the center during the study period. Device choice was determined by patient profile, anatomical considerations, and availability, and outcomes were not analyzed according to specific manufacturer or model. Industry representatives were not involved in patient selection, data collection, or outcome assessment. Device choice was determined by patient profile and availability, but programming was individualized with titration of amplitude, pulse width, and frequency parameters according to clinical response. Rechargeable and non-rechargeable implantable pulse generators were used depending on patient preference and anatomical considerations. Post-implant, patients were systematically reviewed by specialized nurses and pain physicians to optimize programming and troubleshoot technical issues. Reprogramming and minor parameter adjustments formed part of routine post-implant care; however, the number and timing of reprogramming sessions were not systematically recorded and were therefore not included in the present analyses.

### 2.3. Data Collection

Baseline demographic and clinical variables were obtained from electronic health records, including age, sex, pain etiology, history of spine surgery, and pain phenotype (neuropathic vs. mixed). Patient-reported outcomes included pain intensity (VAS) [32], neuropathic pain (DN4) [33], and health-related quality of life (EQ-5D) [34]. Validated Italian versions of DN4 and the EQ-5D visual analogue scale were used in accordance with licensing and local clinical practice. Outcomes were collected prospectively at baseline and at 3, 6, 12, 18, and 24 months post-implantation. Individuals with no follow-up data after implantation were excluded from the analyses, whereas patients with partial follow-up were retained using full-information maximum likelihood estimation. All patients provided written informed consent for participation. Participants were not financially compensated, as data were collected as part of routine clinical care within the SCS Pathway. The study protocol was reviewed and approved by the local Ethics Committee of Comitato Etico Locale (CEL) Palermo 1 (IRB approval, verbale N 23, 19 September 2024).

### 2.4. Statistical Analysis

Analyses were conducted using R (version 4.1.2) and the lme4 package [35]. Descriptive variables and clinical characteristics were summarized using means and standard deviations for continuous variables and frequencies and percentages for categorical variables. MLMs with full information maximum likelihood estimation were used to assess outcomes (VAS, DN4, and EQ-5D) following SCS. The data were organized hierarchically as repeated measurements nested within individual participants across time, thereby controlling for within-subject correlations. Missing data were minimal, with one participant discontinuing after the 12-month follow-up and two additional participants withdrawing after 18 months. This modeling approach allowed for inclusion of all participants, even when certain individuals left the study early.

Time was modeled as a continuous variable using a second-order (quadratic) polynomial to capture non-linear trajectories in longitudinal outcomes. To assess longitudinal changes, independent MLMs were employed for each outcome. Each model included a random intercept for patients and fixed effects for the linear and quadratic time terms, with covariates for age and gender. Additional fixed effects were tested for their contribution to model fit, including pain type, history of spine surgery, and the presence of comorbidities. Model fit was assessed using Akaike Information Criterion (AIC) and likelihood ratio tests. For each outcome, model estimates, standard errors, corresponding *p*-values, and effect sizes from the best-fitting model were reported to evaluate statistical significance.

In addition to these primary analyses, Table A1, Table A2 and Table A3 included descriptive pairwise comparisons between baseline and each follow-up time point, as well as between consecutive time points. These comparisons were conducted using paired *t*-tests and are presented for illustrative purposes to complement the longitudinal model results.

## 3. Results

The study included 93 participants who were assessed for SCS, of whom 85 were deemed eligible for the procedure. Of these, 9 patients did not proceed to permanent implantation due to an unsuccessful or inconclusive trial, while 76 experienced a successful implantation, contributing a total of 452 observations across six different time points. The mean age of participants was 67.3 ± 10.3 years with 39.5% (30/76) being female. The most common chronic pain diagnoses were post-surgical pain syndrome (42.1%, 32/76) and chronic back and leg pain (40.8%, 31/76). A smaller share of participants was diagnosed with neuropathic pain syndrome (9.2%, 7/76), complex regional pain syndrome (6.6%, 5/76), and idiopathic pain syndrome (1.3%, 1/76). Additionally, 42.1% (32/76) of participants had a history of spine surgery, and 78.9% (60/76) experienced neuropathic pain (Table 1). With respect to baseline pharmacological management, most patients were receiving at least one neuropathic pain medication at the time of SCS evaluation, typically anticonvulsants (pregabalin or gabapentin) and/or tricyclic antidepressants, frequently combined with weak or strong opioids and NSAIDs. Only a minority of patients were managed without any specific neuropathic agent, reflecting the refractory nature of pain in this cohort despite multimodal medical therapy.

Responder analyses showed that 65.8% of patients (50/76) achieved ≥50% reduction in VAS at three months, with 52.6% (40/76) maintaining this response at 24 months. When using the more conservative threshold of ≥30% improvement, over 80% of patients met criteria at all time points. Withdrawal was minimal, with three participants lost to follow-up after 12–18 months; no patient required permanent device explantation due to inefficacy within the study window. Complications were rare and consisted primarily of minor lead migration (n = 2) and localized wound irritation (n = 1), all managed conservatively without need for reoperation.

Safety outcomes in this cohort were highly favorable. The overall complication rate (3.9%) was lower than rates reported in multicenter registries, where adverse events typically occur in 5–10% of cases [33]. Importantly, no device explantations for inefficacy or infection occurred within the 24-month observation period, underscoring the robustness of patient selection and follow-up pathways.

**Figure 1:**
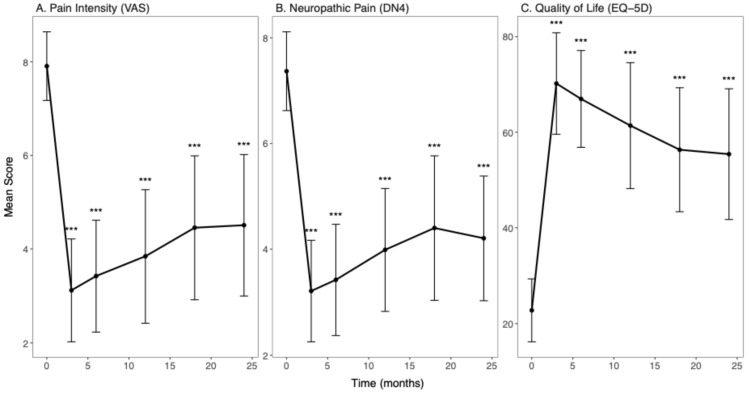
Longitudinal changes in patient-reported outcomes following SCS. (**A**) Mean pain intensity scores measured using VAS. (**B**) Mean neuropathic pain scores using DN4. (**C**) Quality of life scores using EQ-5D. Measurements are shown at baseline (time 0) and at 3, 6, 12, 18, and 24 months post-procedure. Error bars represent standard deviations. Asterisks indicate statistically significant differences at each post-procedure timepoint compared to baseline, based on independent MLMs; *** denotes *p* < 0.001.

Mean pain score decreases were observed in both pain measurements (Figure 1A,B and Table A1 and Table A2). The mean VAS pain score among SCS participants decreased from 7.91 ± 0.73 at baseline to 3.12 ± 1.10 at the 3-month follow-up, representing the largest decrease observed of 61% (*p* < 0.001). While VAS scores showed slight increases, thereafter, decreases relative to baseline persisted over time, with scores of 3.42 ± 1.19 at 6 months (57% decrease; *p* < 0.001), 3.84 ± 1.42 at 12 months (51% decrease; *p* < 0.001), 4.45 ± 1.54 at 18 months (44% decrease; *p* < 0.001) and 4.51 ± 1.51 at 24 months (43% decrease; *p* < 0.001). Similarly, the mean DN4 score decreased from 7.37 ± 0.75 at baseline to 3.21 ± 0.96 at the 3-month follow-up, with a decrease of 56% (*p* < 0.001). Decreases relative to baseline remained substantial, with scores of 3.42 ± 1.05 at 6 months (54% decrease; *p* < 0.001), 3.99 ± 1.16 at 12 months (46% decrease; *p* < 0.001), 4.40 ± 1.37 at 18 months (40% decrease; *p* < 0.001) and 4.21 ± 1.18 at 24 months (43% decrease; *p* < 0.001).

In parallel, mean quality of life (EQ-5D) score increases were observed relative to baseline (Figure 1C and Table A1). The mean EQ-5D score among SCS participants increased from 22.76 ± 6.55 at baseline to 70.20 ± 10.63 at the 3-month follow-up, representing a 208% increase (*p* < 0.001). Although scores declined slightly in following months, substantial improvements relative to baseline were sustained, with scores of 66.97 ± 10.14 at 6 months (194% increase; *p* < 0.001), 61.38 ± 13.18 at 12 months (170% increase; *p* < 0.001), 56.33 ± 12.98 at 18 months (147% increase; *p* < 0.001), and 55.41 ± 13.69 at 24 months (143% increase; *p* < 0.001).

Quadratic models showed a better overall fit for all outcomes, as indicated by lower AIC/BIC values and significant likelihood ratio tests (Table A2). The resulting trajectory was characterized by a marked improvement between baseline and the three-month follow-up, followed by a gradual attenuation and subsequent stabilization over time.

When age, gender, comorbidities, pain type, and history of spine surgery were added as covariates, none consistently predicted changes in VAS, DN4, or EQ-5D across follow-up (Table A3). The only exception was a modest association between prior spine surgery and higher EQ-5D scores (*p* = 0.021). Given the relatively homogeneous and highly selected nature of the cohort, together with the sample size, these exploratory findings should be interpreted as hypothesis-generating rather than conclusive subgroup effects.

## 4. Discussion

In this real-world cohort, SCS was associated with large and statistically significant improvements in pain intensity (VAS), neuropathic pain (DN4), and health-related quality of life (EQ-5D VAS) through 24 months post-implant. These findings are consistent with pivotal randomized controlled trials, including PROCESS [11] and SENZA-RCT [6], which demonstrated that SCS provides superior outcomes compared to conventional medical management. Similarly, the Evoke trial confirmed durable improvements with closed-loop systems over long-term follow-up [7]. The magnitudes of VAS and EQ-5D changes in our cohort fall within the ranges observed in these RCTs, thereby reinforcing the external validity of SCS in real-world settings.

Beyond efficacy, the structured clinical pathway used in our center may have contributed to the high proportion of sustained responders. Multidisciplinary assessment standardized psychosocial screening, and a mandatory trial period mirror recommendations from European consensus guidelines [10,16], supporting the importance of harmonized referral frameworks to ensure optimal outcomes.

The high responder rates and absence of explants for inefficacy in this cohort are more favorable than those reported in some multicenter registries and FBSS-focused series. Several factors may explain these findings. First, the SCS Pathway applies stringent biomedical and psychosocial criteria, and patients with uncontrolled psychiatric conditions or unrealistic expectations are not offered a trial. Second, the use of a 7–14 day screening trial with explicit success thresholds (≥50% pain reduction and/or meaningful functional gains) likely filtered out borderline candidates before permanent implantation. Third, all procedures were performed in a single high-volume center with dedicated programming support and regular nurse-led and physician follow-up, which may have facilitated early detection and correction of technical issues before they resulted in treatment failure. Together, these elements suggest that the combination of careful selection, structured trialing, and close follow-up can substantially enhance long-term SCS effectiveness in routine practice, although external validation in other settings is needed.

The largest improvements were observed at the three-month follow-up, which represents the first scheduled post-implant assessment in this pathway. Although earlier changes could not be captured due to the predefined measurement intervals, the three-month visit showed the peak improvement within the available data, followed by a modest attenuation and subsequent stabilization of outcomes over time.

According to IMMPACT recommendations, ≥30% and ≥50% pain reductions are regarded as “moderately important” and “substantial,” and ~2 points on 0–10 scales is often perceived as clinically important [25,26]. Interpreting our mean changes against these benchmarks, the magnitude of VAS reductions across all time points suggests that many patients likely achieved clinically meaningful pain relief. Parallel reductions on DN4 indicate improvement in neuropathic pain features, complementing prior evidence that SCS can modulate neuropathic-like symptomatology in persistent spinal pain and related syndromes [1,2,3,6,11,18]. The EQ-5D gains—largest at three months and sustained thereafter—support a broad impact on health status, with changes exceeding typical minimally important differences reported in the literature [27]. Strengths of this study include the 24-month observation window in routine care and systematic data capture at predefined time points.

These findings also resonate with prior multicenter registries, which have shown durable improvements in both pain and function post-implant [6,7,11,12,13]. PROCESS and SENZA-RCT demonstrated sustained improvements up to 12 months, which are consistent with the magnitude and trajectory of early benefit seen in our cohort. The Evoke trial further confirmed durable closed-loop outcomes up to 36 months, paralleling the long-term stability we observed, despite partial attenuation after month 3 [6,7,11,12,13]. Importantly, the present data provide a structured perspective by embedding outcomes within a predefined clinical pathway [16,17]. The consistency of reductions in both nociceptive and neuropathic pain domains highlights the multimodal action of SCS and underscores its role in conditions traditionally considered refractory [1,2,3,25]. From a practical standpoint, these results advocate for integration of standardized referral instruments such as the RAND/UCLA Appropriateness Method [19] or clinician-facing e-health decision-support tools [16,17,18,24] into everyday practice. Such tools may reduce unwarranted variability and ensure equitable access to neuromodulation [16,25].

Future directions should focus on harmonizing these pathways at a national and international level, combining clinical expertise with digital platforms capable of real-time patient monitoring [16,17,25]. Artificial intelligence and predictive modeling may soon allow individualized forecasting of treatment response, thus optimizing resource allocation [30]. Furthermore, the incorporation of health economic endpoints and patient-reported outcomes beyond pain, such as work productivity and social participation, will enrich our understanding of the broader value of SCS in chronic pain care [18,27].

Limitations must also be acknowledged. This was a single-center study without a control group, which restricts causal inference, and the regional referral pattern may constrain generalizability and introduce referral bias. Residual confounding is possible as there was no randomized comparator arm. Nevertheless, the structured nature of the SCS Pathway, with standardized assessment and follow-up, strengthens internal validity and offers a model that could be adapted and tested in other healthcare settings. Additional limitations include the heterogeneity of pain diagnoses, which—although aligned with current SCS indications—may introduce clinical variability. Earlier improvements (0–3 months) could not be captured due to predefined measurement intervals. Psychosocial scores (HADS/PCS) were used only for clinical screening and were not available for research analysis. Although pain etiologies differed, all conditions reflected neuropathic or mixed-mechanism chronic pain syndromes recognized as appropriate indications for SCS.

Looking forward, integration of digital health platforms and artificial intelligence could further refine patient selection and long-term monitoring. Predictive algorithms may enable individualized forecasts of treatment response, enhancing efficiency and resource allocation [33]. Additionally, inclusion of health economic endpoints—such as reduced healthcare utilization, return-to-work rates, and improved social participation—will be essential to demonstrate the full societal value of SCS [18].

## 5. Conclusions

In this single-center, real-world cohort embedded in a standardized clinical pathway, spinal cord stimulation was associated with sustained and clinically meaningful improvements in pain intensity, neuropathic symptoms, and health-related quality of life over 24 months. The high responder rates, low complication rate, and absence of explants for inefficacy underscore the importance of rigorous multidisciplinary assessment, structured trialing, and systematic follow-up in everyday practice. These findings support spinal cord stimulation as an effective long-term option for patients with refractory neuropathic or mixed-mechanism pain when delivered within a structured pathway and provide a benchmark for future multicenter studies aimed at validating and refining standardized selection and follow-up frameworks.

## Figures and Tables

**Table 1:** Demographic and Clinical Characteristics.

Characteristic	Total (*N* = 76)
Age in years, M (mean)	67.31 (10.31)
Gender, n (%)	
Male	46 (60.5%)
Female	30 (39.5%)
Chronic pain diagnosis, n (%)	
Chronic back and leg pain	31 (40.8%)
Complex regional pain syndrome	5 (6.6%)
Idiopathic pain syndrome	1 (1.3%)
Neuropathic pain syndrome	7 (9.2%)
Post-surgical pain syndrome	32 (42.1%)
Previous spine surgery, n (%)	
Yes	32 (42.1%)
No	44 (57.9%)
Pain type, n (%)	
Mixed	16 (21.1%)
Neuropathic	60 (78.9%)
Baseline neuropathic pain medication *, n (%) Yes: 75 (98.7%) No: 1 (1.3%)	
Major comorbidities, n (%)	
Cardiovascular	14 (18.4%)
Metabolic	3 (3.9%)
Neurological	5 (6.6%)
Cardiometabolic Multimorbidity	4 (5.3%)
No comorbidities	50 (65.8%)

* Neuropathic pain medication included anticonvulsants (pregabalin, gabapentin), tricyclic antidepressants, opioids, and NSAIDs, alone or in combination. Cardiovascular includes heart-related conditions only (hypertension and heart disease). Metabolic includes diabetes mellitus and hypothyroidism. Neurological includes stroke and transient ischemic attacks. Cardiometabolic multimorbidity includes heart disease + arterial hypertension, or diabetes mellitus, or PM.

## Data Availability

The data presented in this study are available on request from the corresponding author due to privacy and ethical restrictions.

## Appendix A

**Table A1:** Mean pain scores, standard deviations and *p*-values over time following SCS treatment.

Variable	Count	Mean	SD	*p*-Value Relative to Baseline	*p*-Value Relative to Previous Timepoint
VAS					
Baseline	76	7.91	0.73	--	--
3 months post-procedure	76	3.12	1.10	<0.001	<0.001
6 months post-procedure	76	3.42	1.19	<0.001	<0.001
12 months post-procedure	76	3.84	1.42	<0.001	<0.001
18 months post-procedure	75	4.45	1.54	<0.001	<0.001
24 months post-procedure	73	4.51	1.51	<0.001	0.29
DN4					
Baseline	76	7.37	0.75	--	--
3 months post-procedure	76	3.21	0.96	<0.001	<0.001
6 months post-procedure	76	3.42	1.05	<0.001	<0.001
12 months post-procedure	76	3.99	1.16	<0.001	<0.001
18 months post-procedure	75	4.40	1.37	<0.001	<0.001
24 months post-procedure	73	4.21	1.18	<0.001	0.02
EQ-5D					
Baseline	76	22.76	6.55	--	--
3 months post-procedure	76	70.20	10.63	<0.001	<0.001
6 months post-procedure	76	66.97	10.14	<0.001	<0.001
12 months post-procedure	76	61.38	13.18	<0.001	<0.001
18 months post-procedure	75	56.33	12.98	<0.001	<0.001
24 months post-procedure	73	55.41	13.69	<0.001	0.28

**Table A2:** Comparison of model fit statistics for linear versus quadratic time effects.

Model	AIC	BIC	LRT χ^2^	LRT *p*-Value
**VAS**				
Linear time effect	1902.60	1939.60		
Quadratic time effect	1756.00	1797.10	148.65	<0.001 ***
**DN4**				
Linear time effect	1769.80	1806.80		
Quadratic time effect	1654.30	1695.50	117.47	<0.001 ***
**EQ-5D**				
Linear time effect	3945.30	3982.30		
Quadratic time effect	3802.60	3843.70	144.65	<0.001 ***

*** *p* < 0.001.

**Table A3:** Fixed effect estimates and *p*-values from multilevel models with quadratic time terms.

Measure	Estimate	Standard Error	*t* Value	*p*-Value
**VAS**				
Intercept	5.82	0.84	6.96	<0.001 ***
Time, linear component	−9.01	1.55	−5.83	<0.001 ***
Time, quadratic component	20.74	1.54	13.48	<0.001 ***
Gender, Male	−0.02	0.25	−0.07	0.942
Age	−0.01	0.01	−1.09	0.280
Comorbidities present	−0.09	0.26	−0.34	0.735
Previous spine surgery	−0.47	0.25	−1.85	0.068
Neuropathic pain type	−0.17	0.30	−0.56	0.579
**DN4**				
Intercept	4.93	0.70	7.07	<0.001 ***
Time, linear component	−8.80	1.40	−6.29	<0.001 ***
Time, quadratic component	16.33	1.39	11.72	<0.001 ***
Gender, Male	0.02	0.21	0.12	0.907
Age	−0.00	0.01	−0.42	0.675
Comorbidities present	−0.17	0.22	−0.80	0.427
Previous spine surgery	−0.35	0.21	−1.67	0.099
Neuropathic pain type	−0.02	0.25	−0.09	0.929
**EQ-5D**				
Intercept	48.85	7.32	6.67	<0.001 ***
Time, linear component	81.34	15.15	5.37	<0.001 ***
Time, quadratic component	−200.13	15.09	−13.27	<0.001 ***
Gender, Male	−0.54	2.20	−0.25	0.806
Age	0.04	0.11	0.35	0.728
Comorbidities present	1.49	2.29	0.65	0.516
Previous spine surgery	5.19	2.20	2.36	0.021 *
Neuropathic pain type	2.21	2.67	0.83	0.410

* *p* < 0.05; *** *p* < 0.001.

## References

[1] Palmer N., Guan Z., Chai N.C. (2019). Spinal cord stimulation for failed back surgery syndrome—Patient selection considerations. Perioper. Pain Med..

[2] Karri J., Joshi M., Polson G., Agarwal V., Madan A., Ghosh P. (2020). Spinal cord stimulation for chronic pain syndromes: A review of considerations in practice management. Pain Physician.

[3] Celestin J., Edwards R.R., Jamison R.N. (2009). Pretreatment psychosocial variables as predictors of outcomes following lumbar surgery and spinal cord stimulation: A systematic review and literature synthesis. Pain Med..

[4] Lo Bianco G., Papa A., Gazzerro G., Rispoli M., Tammaro D., Di Dato M.T., Vernuccio F., Schatman M. (2021). Dorsal root ganglion stimulation for chronic postoperative pain following thoracic surgery: A pilot study. Neuromodul. Technol. Neural Interface.

[5] Shealy C.N., Mortimer J.T., Reswick J.B. (1967). Electrical inhibition of pain by stimulation of the dorsal columns. Anesth. Analg..

[6] Kapural L., Yu C., Doust M.W., Gliner B.E., Vallejo R., Sitzman B.T., Amirdelfan K., Morgan D.M., Brown L.L., Yearwood T.L. (2015). Novel 10-kHz high-frequency therapy (HF10 therapy) is superior to traditional low-frequency spinal cord stimulation for the treatment of chronic back and leg pain: The SENZA-RCT randomized controlled trial. Anesthesiology.

[7] Mekhail N., Levy R.M., Deer T.R., Kapural L., Li S., Amirdelfan K., Hunter C.W., Rosen S.M., Costandi S.J., Falowski S.M. (2020). Long-term safety and efficacy of closed-loop spinal cord stimulation to treat chronic back and leg pain (Evoke): A double-blind, randomised, controlled trial. Lancet Neurol..

[8] van Hecke O., Torrance N., Smith B.H. (2013). Chronic pain epidemiology and its clinical relevance. Br. J. Anaesth..

[9] Bouhassira D., Lantéri-Minet M., Attal N., Laurent B., Touboul C. (2008). Prevalence of chronic pain with neuropathic characteristics in the general population. Pain.

[10] Deer T.R., Mekhail N., Provenzano D., Pope J., Krames E., Leong M., Levy R.M., Abejon D., Buchser E., Burton A. (2014). The Appropriate Use of Neurostimulation of the Spinal Cord and Peripheral Nervous System for the Treatment of Chronic Pain and Ischemic Diseases: The Neuromodulation Appropriateness Consensus Committee. Neuromodul. Technol. Neural Interface.

[11] Kumar K., Taylor R.S., Jacques L., Eldabe S., Meglio M., Molet J., Thomson S., O’cAllaghan J., Eisenberg E., Milbouw G. (2007). Spinal cord stimulation versus conventional medical management for neuropathic pain: A multicentre randomised controlled trial in patients with failed back surgery syndrome. Pain.

[12] Cruccu G., Garcia-Larrea L., Hansson P., Keindl M., Lefaucheur J., Paulus W., Taylor R., Tronnier V., Truini A., Attal N. (2016). EAN guidelines on central neurostimulation therapy in chronic pain conditions. Eur. J. Neurol..

[13] Dworkin R.H., O’Connor A.B., Kent J., Mackey S.C., Raja S.N., Stacey B.R., Levy R.M., Backonja M., Baron R., Harke H. (2013). Interventional management of neuropathic pain: NeuPSIG recommendations. Pain.

[14] NICE (2008). Spinal Cord Stimulation for Chronic Pain of Neuropathic or Ischaemic Origin. TA159. https://www.nice.org.uk/guidance/ta159.

[15] Thomson S., Huygen F., Prangnell S., De Andrés J., Baranidharan G., Belaïd H., Berry N., Billet B., Cooil J., De Carolis G. (2020). Appropriate referral and selection of patients with chronic pain for spinal cord stimulation: European consensus recommendations and e-health tool. Eur. J. Pain.

[16] Thomson S., Huygen F., Prangnell S., Baranidharan G., Belaïd H., Billet B., Eldabe S., De Carolis G., Demartini L., Gatzinsky K. (2023). Applicability and Validity of an e-Health Tool for the Appropriate Referral and Selection of Patients With Chronic Pain for Spinal Cord Stimulation: Results From a European Retrospective Study. Neuromodul. Technol. Neural Interface.

[17] Taylor R.S., Ryan J., O’Donnell R., Eldabe S., Kumar K., North R.B. (2010). The Cost-effectiveness of Spinal Cord Stimulation in the Treatment of Failed Back Surgery Syndrome. Clin. J. Pain.

[18] Lo Bianco G., Al-Kaisy A., Natoli S., Abd-Elsayed A., Matis G., Papa A., Kapural L., Staats P. (2025). Neuromodulation in chronic pain management: Addressing persistent doubts in spinal cord stimulation. J. Anesth. Analg. Crit. Care.

[19] Sparkes E., Duarte R.V., Mann S., Lawrenc T.R., Raphael J.H. (2015). Analysis of psychological characteristics impacting spinal cord stimulation treatment outcomes: A prospective assessment. Pain Physician.

[20] De La Cruz P., Fama C., Roth S., Haller J., Wilock M., Lange S., Pilitsis J. (2015). Predictors of Spinal Cord Stimulation Success. Neuromodul. Technol. Neural Interface.

[21] Fama C.A., Chen N., Prusik J., Kumar V., Wilock M., Roth S., Pilitsis J.G. (2016). The Use of Preoperative Psychological Evaluations to Predict Spinal Cord Stimulation Success: Our Experience and a Review of the Literature. Neuromodul. Technol. Neural Interface.

[22] Schug S.A., Lavand’homme P., Barke A., Korwisi B., Rief W., Treede R.-D. (2019). IASP Taskforce for the Classification of Chronic Pain. The IASP classification of chronic pain for *ICD-11*: Chronic postsurgical or posttraumatic pain. Pain.

[23] Christelis N., Simpson B., Russo M., Stanton-Hicks M., Barolat G., Thomson S., Schug S., Baron R., Buchser E., Carr D.B. (2021). Persistent Spinal Pain Syndrome: A Proposal for Failed Back Surgery Syndrome and ICD-11. Pain Med..

[24] Dworkin R.H., Turk D.C., Wyrwich K.W., Beaton D., Cleeland C.S., Farrar J.T., Haythornthwaite J.A., Jensen M.P., Kerns R.D., Ader D.N. (2008). Interpreting the clinical importance of treatment outcomes in chronic pain clinical trials: IMMPACT recommendations. J. Pain.

[25] Farrar J.T., Young J.P., LaMoreaux L., Werth J.L., Poole R.M. (2001). Clinical importance of changes in chronic pain intensity measured on an 11-point numerical pain rating scale. Pain.

[26] Pickard A.S., Neary M.P., Cella D. (2007). Estimation of minimally important differences in EQ-5D utility and VAS scores in cancer. Health Qual. Life Outcomes.

[27] Eldabe S., Nevitt S., Griffiths S., Gulve A., Thomson S., Baranidharan G., Houten R., Brookes M., Kansal A., Earle J. (2023). Does a Screening Trial for Spinal Cord Stimulation in Patients With Chronic Pain of Neuropathic Origin Have Clinical Utility (TRIAL-STIM)? 36-Month Results From a Randomized Controlled Trial. Neurosurgery.

[28] Brook R.H., Chassin M.R., Fink A., Solomon D.H., Kosecoff J., Park R.E. (1986). A Method for the Detailed Assessment of the Appropriateness of Medical Technologies. Int. J. Technol. Assess. Health Care.

[29] Lo Bianco G., Cascella M., Li S., Day M., Kapural L., Robinson C.L., Sinagra E. (2025). Reliability, Accuracy, and Comprehensibility of AI-Based Responses to Common Patient Questions Regarding Spinal Cord Stimulation. J. Clin. Med..

[30] Zigmond A.S., Snaith R.P. (1983). The hospital anxiety and depression scale. Acta Psychiatr. Scand..

[31] Darnall B.D., Sturgeon J.A., Cook K.F., Taub C.J., Roy A., Burns J.W., Sullivan M., Mackey S.C. (2017). Development and Validation of a Daily Pain Catastrophizing Scale. J. Pain.

[32] Hawker G.A., Mian S., Kendzerska T., French M. (2011). Measures of adult pain: Visual Analog Scale for Pain (VAS Pain), Numeric Rating Scale for Pain (NRS Pain), McGill Pain Questionnaire (MPQ), Short-Form McGill Pain Questionnaire (SF-MPQ), Chronic Pain Grade Scale (CPGS), Short Form-36 Bodily Pain Scale (SF-36 BPS), and Measure of Intermittent and Constant Osteoarthritis Pain (ICOAP). Arthritis Care Res..

[33] Migliore A., Gigliucci G., Moretti A., Pietrella A., Peresson M., Atzeni F., Sarzi-Puttini P., Bazzichi L., Liguori S., Iolascon G. (2021). Cross cultural adaptation and validation of Italian version of the leeds assessment of neuropathic symptoms and signs scale and pain DETECT questionnaire for the distinction between nociceptive and neuropathic pain. Pain Res. Manag..

[34] Scalone L., Cortesi P.A., Ciampichini R., Belisari A., D’Angiolella L.S., Cesana G., Mantovani L.G. (2013). Italian population-based values of EQ-5D health states. Value Health.

[35] Bates D., Mächler M., Bolker B., Walker S. (2015). lme4 mixed-effects modelling. J. Stat. Softw..

